# Positive and Negative Regulation of Angiogenesis by Soluble Vascular Endothelial Growth Factor Receptor-1

**DOI:** 10.3390/ijms19051306

**Published:** 2018-04-27

**Authors:** Cristina M. Failla, Miriam Carbo, Veronica Morea

**Affiliations:** 1 Istituto Dermopatico dell’Immacolata-IRCCS, 00167 Rome, Italy; 2 Department of Biochemical Sciences “A. Rossi Fanelli”, Sapienza University, 00185 Rome, Italy; miriam.carbo@uniroma1.it; 3 National Research Council of Italy (CNR), Department of Biochemical Sciences “A. Rossi Fanelli”, Institute of Molecular Biology and Pathology c/o, Sapienza University, 00185 Rome, Italy; veronica.morea@uniroma1.it

**Keywords:** Vascular endothelial growth factor receptor, angiogenesis, extracellular matrix

## Abstract

Vascular endothelial growth factor receptor (VEGFR)-1 exists in different forms, derived from alternative splicing of the same gene. In addition to the transmembrane form, endothelial cells produce a soluble VEGFR-1 (sVEGFR-1) isoform, whereas non-endothelial cells produce both sVEGFR-1 and a different soluble molecule, known as soluble fms-like tyrosine kinase (sFlt)1-14. By binding members of the vascular endothelial growth factor (VEGF) family, the soluble forms reduce the amounts of VEGFs available for the interaction with their transmembrane receptors, thereby negatively regulating VEGFR-mediated signaling. In agreement with this activity, high levels of circulating sVEGFR-1 or sFlt1-14 are associated with different pathological conditions involving vascular dysfunction. Moreover, sVEGFR-1 and sFlt1-14 have an additional role in angiogenesis: they are deposited in the endothelial cell and pericyte extracellular matrix, and interact with cell membrane components. Interaction of sVEGFR-1 with α5β1 integrin on endothelial cell membranes regulates vessel growth, triggering a dynamic, pro-angiogenic phenotype. Interaction of sVEGFR-1/sFlt1-14 with cell membrane glycosphingolipids in lipid rafts controls kidney cell morphology and glomerular barrier functions. These cell–matrix contacts represent attractive novel targets for pharmacological intervention in addition to those addressing interactions between VEGFs and their receptors.

## 1. Introduction

During embryo development, cells from mesoderm differentiate into pluripotent hemangioblasts, and then into angioblasts and endothelial cells, in a process called vasculogenesis. Endothelial cells assemble into a primary capillary plexus that forms new capillaries and vessels by angiogenesis, as part of the development of a new mature vascular system. 

Angiogenesis, defined as the growth of new vessels from existing ones, is a main feature of embryonic development, where it is of utmost importance [1]. In fact, blocking embryonic angiogenesis leads to impairment of development and death of the embryo at an early stage, due to the lack of a functional vascular system able to supply nutrients and oxygen and efficiently remove metabolic byproducts. In adult life, angiogenesis is limited to particular physiological cases, such as hair growth, ovarian and endometrial cycles, and wound healing. On the other hand, angiogenesis is associated with a number of pathologies in adults, where tumor-derived or inflammation-driven molecules impair the existing tissue balance between angiogenic inhibitors and activators [2]. This imbalance augments the presence of angiogenic factors, to which endothelial cells respond by starting to migrate and proliferate by sprouting angiogenesis. The newly forming vessel network is also augmented by non-sprouting angiogenesis (intussusception), namely the division of a vessel in two by formation of a cellular septum in the center, and undergoes a fine remodeling that gives rise to a novel vascular system [1]. 

**Figure 1:**
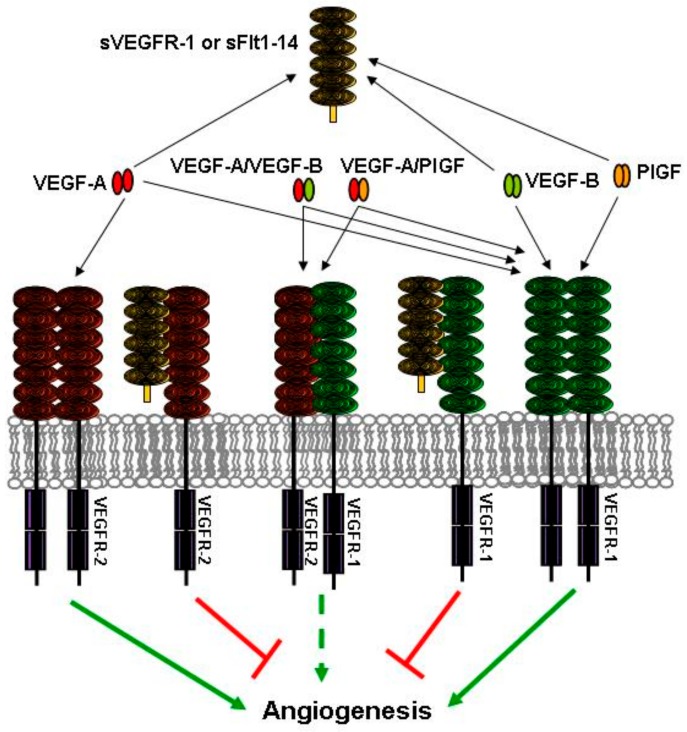
Anti-angiogenic roles of soluble vascular endothelial growth factor receptor (VEGFR)-1 VEGFR-1 isoforms. Soluble VEGFR-1 isoforms, either sVEGFR-1 or soluble fms-like tyrosine kinase (sFlt)1-14, act as “VEGF sinks”. By binding members of the VEGF family, they decrease the amount of growth factors available to interact with the transmembrane tyrosine kinase receptors. In addition, soluble receptor isoforms can reduce VEGF signal transduction by binding to transmembrane receptor monomers and blocking formation of signaling competent receptor homodimers. Receptor heterodimers can also be inhibited by soluble isoforms, thus blocking the signaling of VEGF-A/placenta growth factor (PlGF) or VEGF-A/VEGF-B heterodimers. Interactions that lead to and hamper angiogenesis are indicated by green and red arrows/lines, respectively. The dashed arrow indicates the limited pro-angiogenic activity of receptor heterodimers.

Vascular endothelial growth factor receptor-1 (VEGFR-1), previously known as fms-like tyrosine kinase (Flt-1) [3], is a membrane receptor for different members of the vascular endothelial growth factor (VEGF) family. In particular, VEGFR-1 binds VEGF-A with high affinity, and is the only known tyrosine kinase receptor for VEGF-B and placenta growth factor (PlGF) [4,5]. Initial gene knockout studies showed that VEGFR-1 was essential for development and differentiation of the embryonic vasculature. In fact, embryos in which VEGFR-1 has been knocked-out died in utero between day 8.5 and 9.0 [6]. The defect was later ascribed to an increased outgrowth of endothelial cells and angioblast commitment, which inhibited a proper organization of the embryonal vascular network [7]. In the same work, VEGFR-1 role in vasculogenesis and angiogenesis was ascribed to VEGF-A binding, which determined both suppression of excessive angioblast development and hampering of VEGF-A-mediated signaling. Indeed, VEGFR-1 had been previously proposed to act as a “VEGF-sink”, regulating the amount of VEGF-A available for vascular development through interaction with the other tyrosine kinase receptors VEGFR-2 or VEGFR-3 [8]. In agreement with this hypothesis, mice carrying a homozygous deletion of VEGFR-1 intracellular kinase domain showed correct development of blood vessels [8]. This result indicated that VEGFR-1 had a primary role in embryonic angiogenesis, independent of its tyrosine kinase activity and restricted to its extracellular region. Actually, a differentially spliced form of VEGFR-1 mRNA encoding a soluble receptor variant (sVEGFR-1) was isolated from cultured endothelial cells. sVEGFR-1 comprises the 656 N-terminal residues of the receptor, followed by a specific 30 amino acid tail at its C-terminus. This form is proposed to function as a modulator of VEGF-A-dependent signaling, by forming non-signaling complexes with VEGFR-2 [9]. sVEGFR-1 has been later isolated from different cell lines and demonstrated to act as a naturally-produced VEGF antagonist that inhibits the mitogenic effects of VEGF-family growth factors by functioning as a dominant-negative trapping protein [10]. In fact, addition of sVEGFR-1 can partially rescue the phenotype of VEGFR-1 knockout mice, reducing the levels of VEGFR-2 tyrosine phosphorylation [11]. A second soluble form of VEGFR-1 has been then isolated, generated by alternative splicing downstream of exon 14 and polyadenylation within an Alu element [12,13]. This soluble VEGFR-1, currently named sFlt1-14 or sFlt1-e15a, encodes a C-terminal variant with a polyserine tail, and is produced by non-endothelial cells, such as epithelial, dendritic cells, monocytes/macrophages, and vascular smooth muscle cells. An additional soluble form of VEGFR-1 is generated by proteolytic cleavage of the transmembrane protein in the extracellular portion, adjacent to the transmembrane domain [14]. These latter soluble isoforms have been shown to act as VEGF antagonists as well. Altogether, studies with both animal models and cultured cells support the concept that the main function of soluble VEGFR-1 isoforms, as well as the membrane one, is negative regulation of VEGF signaling (Figure 1) [15]. 

In recent years, VEGF-B and PlGF signaling through transmembrane VEGFR-1 has been clearly correlated with pathological angiogenesis in adults, and both growth factors have been recognized as angiogenic factors, even in the absence of VEGF-A [16,17]. Thus, transmembrane VEGFR-1 transmits a pro-angiogenic signal, and has a function besides that of VEGF-A sequestration or receptor antagonism through dimerization [15]. Additionally, high levels of sVEGFR-1 or sFlt1-14 are found in pathological conditions involved in vascular dysfunction, as discussed below. These observations restrict the hypothesis that VEGFR-1 acts as a VEGF-sink/transmembrane receptor inhibitor to soluble VEGFR-1 isoforms only. Nevertheless, there are still unanswered questions about the assumption that soluble VEGFR-1 isoforms have an exclusively negative role. It is relevant to note that the knockout model in which only the tyrosine kinase domain was deleted [8] had the ability to produce both a transmembrane form of VEGFR-1 without the intracellular domain, and the soluble forms sVEGFR-1 and sFlt1-14. Therefore, the normal mouse phenotype could be ascribed to one of the following: formation of inactive dimers with transmembrane receptors; interaction of soluble isoforms with VEGF-A; and/or interaction of soluble isoforms with other molecules. A later mouse model was generated that lacked both VEGFR-1 tyrosine kinase and transmembrane domain, resulting in the production of soluble isoforms only [18]. About one-half of these mice died as embryos with abnormal blood vessel formation, indicating that the presence of VEGFR-1 extracellular region anchored to the membrane by the transmembrane domain was required to efficiently inhibit VEGF-A pro-angiogenic action. Since VEGF-A binding region is present in the soluble isoforms as well, the highest inhibition of VEGF-A signaling by the transmembrane form can be ascribed, at least in part, to a greater ability to heterodimerize with VEGFR-2, thus reducing VEGF-A signaling mediated by the latter receptor. In agreement with this hypothesis, the same mouse model produced in a different genetic background, and expressing higher VEGFR-2 levels than the previous strain, led to the survival of most animals [18]. This suggests that increased amounts of VEGFR-2 can compensate for a putatively reduced activity of the soluble forms as VEGF inhibitors. However, it could not be ruled out that other differences between the two mouse strains (e.g., in factors involved in cell-to-matrix interactions) contributed to a reversion of the mouse phenotype back to normal.

## 2. Structure of the Soluble Forms of VEGFR-1

Eight VEGFR-1 isoforms are present in the UniProt database [19] at the time of writing. The aforementioned transmembrane VEGFR-1, sVEGFR-1, and sFlt1-14 correspond to isoforms 1, 2, and 3, respectively. Isoform 4 is an additional extracellular soluble protein, shorter than sFlt1-14 and sVEGFR-1 (it encompasses the five N-terminal immunoglobulin (Ig)-like domains, D1–D5) and produced by proteolytic cleavage. Isoforms 5 to 8 map in the intracellular region of the receptor. Isoform 5 comprises the whole tyrosine kinase domain, whereas isoforms 6, 7, and 8 are truncated variants missing different N-terminal regions of the tyrosine kinase domain. The biological significance of isoforms 4 to 8 is still unclear.

**Figure 2:**
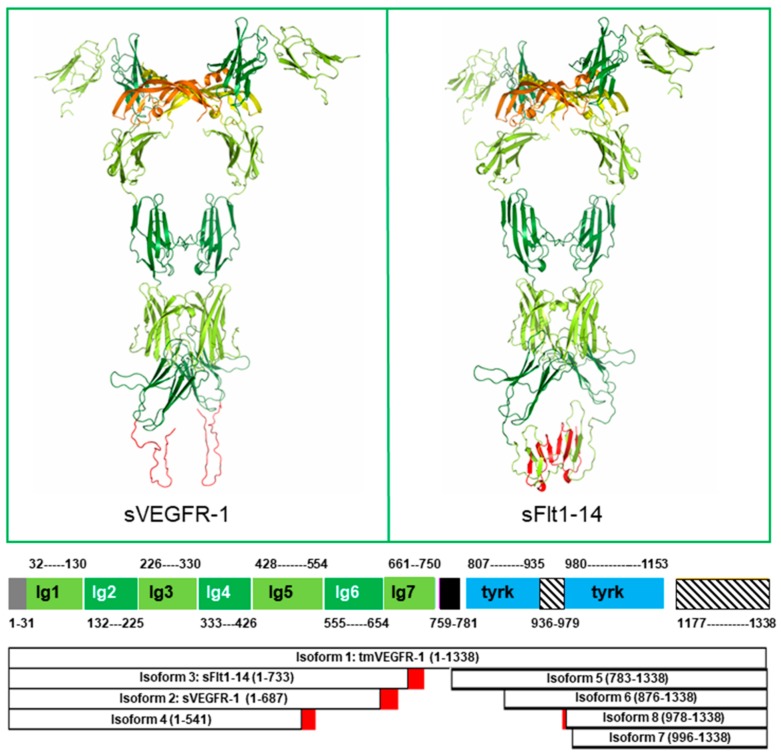
VEGFR-1 structures. Top: Soluble isoforms sVEGFR-1 (**left**) and sFlt1-14 (**right**) are shown by ribbon representation. The 3D structure of Ig-like domains 1–6 (residues 1–654), common to both isoforms, has been experimentally determined in complex with VEGF-A (PDB ID: 5T89, Resolution: 4 Å). VEGF monomers are orange and yellow. VEGFR-1 Ig-like domains 1, 3 and 5 are light green; Ig-like domains 2, 4 and 6 are dark green; residues 657–705 of sFlt-1, whose sequence is identical to transmembrane VEGFR-1 (isoform 1) are light green; the 30 residues C-terminal tail of sVEGFR-1 (657–687) and residues 706–733 of sFlt-1, whose sequence differs from isoform 1 are red. The C-terminal regions of sVEGFR-1 and sFlt1-14 have been modeled by homology using the structure of VEGFR-2 domain 7 (PDB ID: 3KVQ, Resolution: 2.7 Å) as a template, using the molecular graphics program InsightII (Accelrys Software Inc., [26]). However, both these regions are likely to be, at least in the red-colored part, disordered (see text). Bottom: Domain architecture of VEGFR-1 isoforms. VEGFR-1 is color coded as follows: N-terminal signal sequence, grey; Ig-like domains 1, 3, 5 and 7, light green; Ig-like domains 2, 4, and 6, dark green; transmembrane helix, black; tyrosine kinase domain, blue; disordered regions, striped. Isoform sequences that differ from transmembrane VEGFR-1 are red. Boundaries of Ig-like domains 1–6 are defined according to the experimental structure. All the other regions are defined based on a combination of programs for domain assignment (i.e., SMART [27], Pfam [28], Superfamily [29]) and transmembrane helices (i.e., TMHMM [30]) or secondary structure and disordered region predictions (i.e., PsiPred [25]). The length of each segment in the picture is proportional to the number of residues comprised in each domain or region.

High resolution three-dimensional (3D) structures of the second VEGFR-1 Ig-like domain (D2) have been determined by X-ray crystallography and are available from the Protein Data Bank (PDB, www.rcsb.org) [20]. In all these structures, D2 is in complex with one of its growth factor ligands: VEGF-A (PDB ID: 1FLT, Resolution: 1.7 Å) [21]; VEGF-B (PDB ID: 2XAC, Resolution: 2.71 Å) [22]; and PlGF (PDB ID: 1RV6, Resolution: 2.45 Å) [23]. More recently, the three-dimensional (3D) structure of the almost complete VEGFR-1 extracellular region has been determined by employing different techniques, including X-ray crystallography, single-particle electron microscopy, and molecular modeling (PDB ID: 5T89, Resolution: 4 Å) [24] (Figure 2). The structure comprises the six N-terminal Ig-like domains D1–D6 in complex with VEGF-A, and is the largest structure of one member of the VEGFR family reported to date. 

Coordinates of VEGFR-1 Ig-like domain 7 (D7) are not available from the PDB; however, a molecular model of this domain can be built using the experimentally determined structure of the homologous VEGFR-2 D7 (PDB ID: 3KVQ, Resolution: 2.7 Å) as a template. As far as the C-terminal regions of soluble VEGFR-1 isoforms are concerned, the specific 30 residues C-terminal tail of sVEGFR-1 is predicted to be disordered by PsiPred [25] (Figure 2). In the case of sFlt-1, the region following D6 is likely to be disordered as well. In fact, of the seven β-strands that form the two β-sheets packed together in Ig-like domains, three are lost due to the replacement of the 706–733 region by a low complexity sequence (20 out of 28 residues are serine), and another one is missing due to the absence of residues 734–750 (Figure 2). 

Analysis of the aforementioned structures allowed VEGFR-1 residues involved in the following interactions to be identified: D2 residues interacting with VEGF-A, VEGF-B, or PlGF; D3 residues interacting with VEGF-A; D4 and D5 residues involved in homotypic interactions between receptor monomers, which occur downstream the growth factor binding region (Table 1). By superimposing the structure of the D2 domain in complex with VEGF-B or PlGF to the D2 domain in the D1–D6 structure, D3 residues interacting with VEGF-B and PlGF could be inferred (Table 1). Finally, homotypic interactions involving D7 domains, which are not present in the structures, were inferred from a homology model built using as template the 3D structure of the homologous VEGFR-2 domain (PDB ID: 3KVQ, Resolution: 2.7 Å) [31] (Table 1).

Knowledge of VEGFR-1 residues involved in essential interactions is a fundamental requirement to rationally design molecules able to modulate angiogenesis and other biological functions mediated by VEGFR-1 (see below). Indeed, several peptides mapping on the surface of VEGFR-1 D2 structure were designed and shown to be endowed with pro- or anti-angiogenic activity, which was exerted by mimicking or interfering with VEGFR-1 interactions [32,33,34].

## 3. Expression of Soluble Forms of VEGFR-1

The molecular mechanisms regulating alternative splicing process that gives raise to different soluble forms of VEGFR-1 have been investigated. Differential splicing involves *cis*-regulatory elements in the sVEGFR-1 mRNA, and has a major processing site in intron 13 [35]. While expression of transmembrane VEGFR-1 and VEGF-A are increased by hypoxia [36,37], it is not clear whether sVEGFR-1 expression is downregulated or upregulated by reduced oxygen concentration. In endothelial cells, sVEGFR-1 was reported to be downregulated by hypoxia through a mechanism that did not directly involve the hypoxia-inducible factor (HIF)-1α [38]. On the other hand, hypoxia augments the amount of sVEGFR-1 produced by macrophages/monocytes after exposure to granulocyte-macrophage colony-stimulating factor (GM-CSF), and this induction is dependent on HIF-2α with no contribution by HIF-1α [39]. Another study indicated that hypoxic conditions increase secretion of sFlt1-14 by a signaling pathway that comprises activation of the growth arrest and DNA damage-inducible 45a (Gadd45a) factor and p38 phosphorylation [40]. At variance with sVEGFR-1, which has been detected in most tissues, sFlt1-14 is mainly produced in the placenta by cytotrophoblasts [41]. In this cell type, exposure to hypoxia increases both sFlt1-14 and sVEGFR-1 mRNA, probably through stabilization of HIF-1α protein [41]. Other drugs inhibit sFlt1-14 production by cytotrophoblasts using various pathways. Ouabain, a cardiac glycoside, downregulates sFlt1-14 production through a HIF1α/heat-shock protein 27 (HSP27) pathway [42]. Aspirin decreases sFlt1-14 expression by inhibition of cyclooxygenase 1 [43] whereas metformin reduces sFlt1-14 secretion possibly by inhibiting the mitochondrial electron transport chain [44]. Hydroxylases or demethylases can also influence sVEGFR-1/sFlt1-14 expression. In fact, Jumonji domain-containing protein 6 regulates VEGFR-1 mRNA splicing by interacting with and hydroxylating splicing factor U2AF65. Since this hydroxylating reaction depends on oxygen concentration, hypoxia results in augmented sVEGFR-1/sFlt1-14 levels [45]. Differential methylation has been found in a CpG island of VEGFR-1 promoter region in smoker’s lung macrophages compared to cells from non-smoking individuals [46]. This results in increased levels of all three VEGFR-1 isoforms, i.e., the transmembrane, sVEGFR-1, and sFlt1-14, with sFlt1-14 being the most upregulated. Treatment with phorbol myristic acid, an activator of protein kinase C, increases the abundance of sVEGFR-1 mRNA and protein in endothelial cells [35]. Importantly, VEGF-A itself can stimulate the expression of sVEGFR-1 by activating VEGFR-2 and protein kinase C [14].

Besides GM-CSF-mediated induction of sVEGFR-1 in human monocytes [47], at least two other cytokines, interleukin-4 in macrophages and interleukin-6 in endothelial cells, increase sVEGFR-1/sFlt1-14 secretion [38,48]. 

In contrast with what happens for VEGF-A expression, a peptide that mimics the mRNA regulatory protein AUF1/heterogeneous nuclear ribonucleoprotein (hnRNP) D stimulates soluble VEGFR-1 isoform expression in macrophages [49]. In a similar way, and different from VEGF-A induction, mechanical forces downregulate sVEGFR-1 expression by human chondrocytes [50]. In summary, soluble receptor isoforms and their ligands often appear to be inversely regulated. 

As far as post-translation modifications are concerned, it should be considered that VEGFR-1 soluble forms are highly glycosylated, and inhibition of glycosylation, as well as of intracellular trafficking, reduces secretion of these forms [12]. Soluble VEGFR-1 isoforms are also heparin binding proteins, and they are stored in the vessel walls or in the placenta through heparin binding. Both heparin and heparanase treatment release soluble isoforms from their storage matrix and release them into circulation [51]. Thus, heparanase inhibition could be an alternative therapeutic target in pathologies, where high amounts of soluble isoforms are present in the patient blood.

## 4. sVEGFR-1 and Vessel Sprouting

Sprouting angiogenesis consists of migration of endothelial cells from a parental vessel to give rise to new vascular structures. Endothelial cells send out filopodia and migrate away from the parent vessel without breaking contact with other endothelial cells, so that the sprout comprises several cells. Moreover, endothelial cell division takes place, at the same time, behind the sprout leading tip [52]. Sprouting angiogenesis is modulated by different angiogenic factors mainly belonging to the VEGF, platelet-derived growth factor (PDGF), and angiopoietin families [53].

A confocal time-lapse imaging study of VEGFR-1 knockout embryonic stem cells revealed that sVEGFR-1 is important in modulating endothelial cell migration and vascular sprouting during development [54]. The proposed mechanism is based on the production of sVEGFR-1 by endothelial cells during vessel morphogenesis, and the formation of a VEGF-A gradient through sVEGFR-1/VEGF-A interaction, which sequesters VEGF-A and locally inactivates VEGFR-2 signaling. Endothelial cells derived from VEGFR-1 knockout embryonic stem cells have an increased mitotic index, and defects in sprouting and branch formation [54,55]. This phenotype could be rescued by reintroducing either transmembrane VEGFR-1 or sVEGFR-1; however, only sVEGFR-1 mends the branching morphogenesis defect [56]. Therefore, sVEGFR-1 has been proposed to act as a guidance molecule in vessel sprouting. By inactivating VEGF-A at either side of the sprout, sVEGFR-1 provides a VEGF-A-rich corridor for the emerging vessel, maintaining it in the proper direction [56]. Interestingly, vessel morphogenetic defects of VEGFR-1 knockout embryonic stem cells could be rescued through inactivation of the Notch pathway, which is also activated by VEGF-A [57]. 

## 5. Soluble Forms of VEGFR-1 in Human Pathologies

Besides their role in tumor development and progression, which have been described elsewhere [58,59], soluble VEGFR-1 isoforms have been related with different pathological conditions. In 2006, the group of J. Ambati described, for the first time, the involvement of sVEGFR-1 in preserving cornea avascularity through specific inhibition of VEGF-A [60]. Other works demonstrated that the avascular privilege of retina photoreceptor layer depends on sVEGFR-1-mediated inactivation of VEGF-A, as well. Reduced sVEGFR-1 expression was observed in patients suffering from age-related macular degeneration, where retina angiomatous proliferation and choroidal neovascularization are present [61]. Interestingly, reduced serum sVEGFR-1 occurred in patients with neovascular age-related macular degeneration, with respect to earlier manifestations of the same pathology or healthy individuals [62]. During the wound healing process, sVEGFR-1 levels in wound fluid are initially low, similar to serum level; they increase during granulation tissue formation, and decrease again at wound closure. However, in the fluid of chronic non-healing wounds, higher amounts of sVEGFR-1 are present, and contribute to sustaining chronic ulceration [63]. Soluble VEGFR-1 isoforms have been found in the blood of pregnant women, but not in nonpregnant women and men [64], and excess circulating soluble receptors contribute to the pathogenesis of the pregnancy disorder pre-eclampsia [65,66]. It is not clear which one of the two isoforms is involved, but a main role is surely played by sFlt1-14, because it is selectively expressed in the placenta, and could be considered as a placenta-specific protein [41]. Increased sFlt1-14 produced by the placenta sequesters VEGF-A and PlGF, causing generalized endothelial dysfunction and altered neutrophil activation and migration [67]. This results in the clinical features of pre-eclampsia, such as hypertension, proteinuria, and glomerular endotheliosis [66]. It is now well established that increased circulating levels of sFlt1-14 [68] and, even more, of the sFlt1-14/PlGF ratio [69], can be used to predict pre-eclampsia development. Occurrence of hypertension in pre-eclampsia patients could be mediated by sFlt1-14 inhibition of VEGF-A and consequent reduced levels of nitric oxide and augmented amount of endothelin-1, a potent vasoconstrictor [70]. sVEGFR-1 has been linked to endothelial cell function in the kidney. Renal glomerular microvasculature is particular susceptible to local availability of VEGF-A [71]. Plasma levels of sVEGFR-1 are higher in patients with chronic kidney disease, and correlate with cardiovascular morbidity and heart failure [72,73]. Finally, soluble VEGFR-1 isoforms have been linked to different states of inflammation. Increasing levels of sVEGFR-1 are present in the blood of patients with sepsis, and may represent a potential new marker of disease severity [74]. Augmented plasma VEGF-A and sVEGFR-1 are present in liver cirrhosis patients compared to healthy individuals [75], and serum levels of sVEGFR-1 represent a predictor of endothelial dysfunction and activation of coagulation in acute pancreatitis [76]. Monocyte activation is important for a proper immune response. Monocytes are able to bind immunoglobulins on target cells by their Fc-γ receptor. This interaction stimulates monocytes to release pro-inflammatory cytokines, and initiate phagocytosis and antibody-mediated cellular cytotoxicity. Interestingly, immunoglobulin-Fc-γ receptor engagement also stimulates monocytes to secrete soluble VEGFR-1 isoforms [77]. The secreted isoforms, by blocking VEGF-A signaling, contribute to reducing inflammatory angiogenesis, as well as interstitial pressure and edema. 

Regarding atherosclerosis, no clear data are available at present. On the one hand, reduced levels of sVEGFR-1 and higher PlGF/sVEGFR-1 ratio are associated with accelerated progression of atherosclerosis in patients suffering from renal dysfunction and in an experimental animal model [78]. On the other hand, high levels of sVEGFR-1 in hypertensive patients are an indicator of the progression of carotid intima-media thickness, a marker of atherosclerosis [79]. Surely, additional studies are required to fully understand the role and reveal the molecular mechanism of action of sVEGFR-1 and sFlt1-14 in most pathologies.

## 6. Soluble VEGFR-1 Isoforms in the Extracellular Matrix

**Figure 3:**
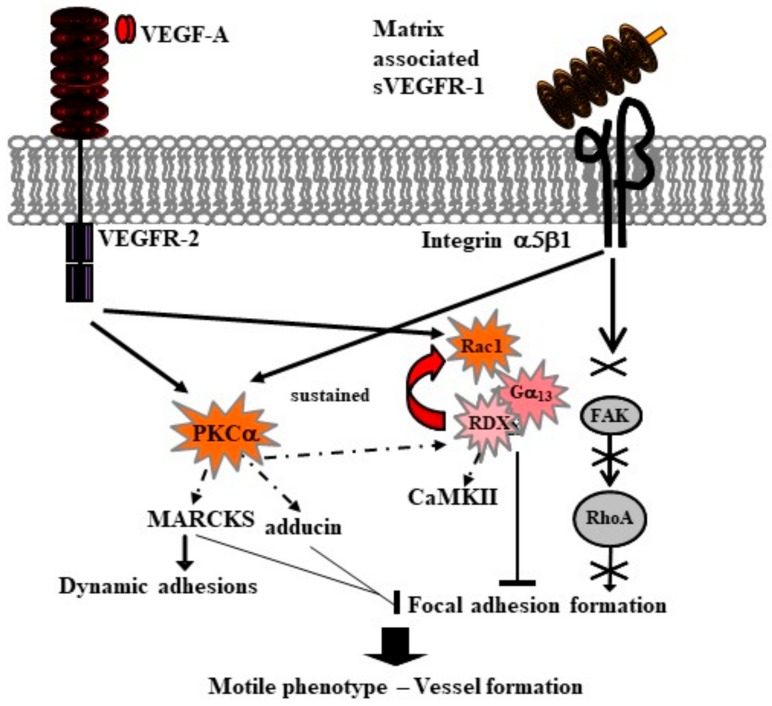
Molecular signals of motility are activated by endothelial cell adhesion to matrix sVEGFR-1 through α5β1 integrin. Protein kinase C alpha (PKCα) phosphorylation by VEGFR-2 and/or α5β1 integrin (arrows) modulates both expression and phosphorylation (dotted arrow) of its substrates adducin, myristoylated alanine-rich protein kinase C substrate (MARCKS), and radixin (RDX) (see text). VEGFR-2 starts a signaling loop that involves the Rho family GTPase Rac1 and the heterotrimeric G protein α13 (Gα13), and is sustained (red arrow) by the activation of radixin, which also phosphorylates the calcium/calmodulin-dependent protein kinase II (CaMKII). Cell adhesion to matrix sVEGFR-1 does not activate the focal adhesion kinase (FAK) signaling (cross and crossed arrows). As a consequence, focal adhesion formation is blocked (bars). MARCKS expression is increased, and non-phosphorylated MARCKS accumulates into and stabilizes dynamic adhesions (black arrow). Thus, endothelial cells acquire a highly motile phenotype that allows them to initiate new vessel formation (thick black arrow).

In the light of observation that not all the biological effects of sVEGFR-1 could be ascribed to the regulation of VEGF signaling, we decided to investigate sVEGFR-1 location in the endothelial cell microenvironment. In 2003, we showed, for the first time, that sVEGFR-1 is deposited by cultured endothelial cells in the extracellular matrix, where it determines endothelial cell adhesion and migration [80]. This activity is mediated by sVEGFR-1 interaction with α5β1 integrin, which is highly involved in angiogenesis. In fact, antagonists of α5β1 integrin inhibit tumor vascularization [81], and α5 subunit knockout is associated with reduced formation of vessel structures [82], and with vascular and cardiac defects [83]. sVEGFR-1 interaction with α5β1 integrin is independent of the presence of fibronectin or other matrix proteins that interact with integrins through an RGD sequence motif. Additionally, neither VEGF-A or PlGF mediate sVEGFR-1 binding to α5β1 integrin. Notably, endothelial cells spread weakly on sVEGFR-1-coated plates, displaying a cytoskeleton organization different from that stimulated by adhesion on fibronectin [80]. To elucidate the molecular determinants of sVEGFR-1/integrin interaction, we initially identified receptor D2 as a main site of interaction. Subsequently, we isolated a seven-amino acid-long peptide mapping on this domain (peptide 12) which efficiently inhibits sVEGFR-1 interaction with α5β1 integrin [84]. Moreover, peptide 12 supports, by itself, α5β1 integrin-mediated endothelial cell adhesion, migration, and vessel formation, both in vitro and in vivo [84]. Following fibronectin binding, α5β1 integrin initiates a signal that leads to phosphorylation of the focal adhesion kinase (FAK) and of the adaptor Src-homology 2 domain-containing (Shc) protein [85]. Interestingly, peptide 12 interaction with α5β1 integrin does not activate the FAK pathway, and induces only slight phosphorylation of Shc [84]. Further work from our group revealed that sVEGFR-1, as well as peptide 12 interaction with α5β1 integrin, produces a dynamic, motile phenotype in endothelial cells by activation of different molecular pathways. After endothelial cell adhesion on sVEGFR-1, VEGFR-2 and/or α5β1 integrin initiate a signal that leads to activation of protein kinase C alpha (PKCα) and its substrates adducin, myristoylated alanine-rich protein kinase C substrate (MARCKS), and radixin [86]. Phosphorylation reduces adducin activity, thus contributing to delaying focal adhesion assembly. Formation of focal adhesion is not stimulated by FAK phosphorylation either, as sVEGFR-1 binding to α5β1 integrin did not initiate FAK signaling [86]. MARCKS protein levels are increased after adhesion to matrix sVEGFR-1, but MARCKS is not efficiently phosphorylated. Non-phosphorylated protein accumulates into structures, called dynamic adhesions [87], contributing to stabilizing these structures and prompting cell movements [88]. Both α5β1 integrin and VEGFR-2 are known to regulate cell adhesion and migration through the Rho family GTPases [89]. Only GTPase Rho family GTPase Rac1 is activated in cells adherent to sVEGFR-1, probably by VEGFR-2 stimulation, since a FAK-mediated switch is absent. However, Rac1 activation persists in time. Continual activation is due to binding to the heterotrimeric G protein α13 (Gα13) that forms a complex with and induces a conformational change of radixin. This, in turn, stimulates Rac1. Moreover, radixin phosphorylates calcium/calmodulin-dependent protein kinase II (CaMKII) [86] (Figure 3). 

The resulting endothelial cell phenotype is highly mobile, allowing sprouting angiogenesis and formation of new vessels [86].

In 2012, the group of S. E. Quaggin found that kidney podocytes produced soluble VEGFR-1 isoforms, and that selective deletion of VEGFR-1 highly affected cytoskeletal reorganization and podocyte morphology, leading to development of proteinuria [90]. Reintroduction of VEGFR-1 extracellular domain, i.e., its soluble forms, rescued the mouse phenotype. Interestingly, normal podocytes adhere to sVEGFR-1/sFlt1-14, but this adhesion is not mediated by integrin binding. Soluble receptors interact with podocyte membrane in a heparin-dependent way and directly bind to the glycosphingolipid ganglioside (GM) 3, a component of lipid rafts [90]. As in the case of α5β1 integrin binding, VEGF-A does not mediate sVEGFR-1/sFlt1-14 direct binding to GM3. Upon adhesion on sVEGFR-1/sFlt1-14, podocytes transduce a signal that comprises phosphorylation of syndecan 1, syndecan 4, and nephrin, and specifically regulates actin cytoskeleton. These interactions are present in pericytes and perivascular cells from various tissues, but not in endothelial cells themselves [90].

Altogether, these data indicate that soluble VEGFR-1 isoforms regulate vascular integrity and movements by binding to molecules different from VEGFs.

## 7. Conclusions

The role of transmembrane VEGFR-1 in the angiogenic process has been underestimated for a long period, probably because of its dispensability as tyrosine kinase receptor during embryonic development. In recent days, transmembrane VEGFR-1 and its selective ligands PlGF and VEGF-B have been highly involved in pathological angiogenesis where they play non-redundant roles [91,92]. Soluble VEGFR-1 isoforms have been clearly implicated in pathological angiogenesis as well, because of their action as VEGF-sinks and/or inhibition of transmembrane VEGFR dimerization and signaling. However, sVEGFR-1/sFlt-1 are likely to have an active role in angiogenesis, which is exerted by binding molecules different from VEGFs. Perivascular cells produce and bind to soluble VEGFR-1 isoforms through GM3 ganglioside, thus controlling actin cytoskeleton dynamics [90]. Since destabilization of pericyte-endothelial interactions is a prerequisite for sprouting angiogenesis and requires alteration of perivascular cell cytoskeleton, as well as modification of adhesion contacts with basement membrane, these findings support the hypothesis that sVEGFR-1/sFlt1-14 are involved in vessel sprouting, taking part in different molecular interactions. Our data indicate that the presence of sVEGFR-1 shifts α5β1 integrin signaling from a classic adhesion pathway to a more dynamic one [86]. Presence of sVEGFR-1 in the endothelial cell microenvironment during vessel sprouting has been clearly shown [55,56], as well as augmented expression of α5β1 integrin [93]. These data suggest that sVEGFR-1/sFlt1-14 participate in vessel sprouting with mechanisms that go beyond VEGF binding. At present, it is not possible to dissect the relative importance of VEGFR-1 interactions with VEGFs and α5β1 integrin, since they are mediated by the same VEGFR-1 D2, even if they involve different amino acid residues. Development of tailored sVEGFR-1 mutants, able to selectively interact with VEGFs or with α5β1 integrin, is required to clarify this point.

In conclusion, new data are required to understand the balance between sVEGFR-1/sFlt1-14 promoting and inhibiting roles in angiogenesis, and to develop novel and effective therapeutic compounds targeting these molecules.

## Figures and Tables

**Table 1:** VEGFR-1 residues involved in interaction with ligands or dimer formation.

**PDB ID: 1FLT**		**PDB ID: 2XAC**		**PDB ID: 1RV6**			
**VEGFR-1 D2**	**VEGF-A**	**VEGFR-1 D2**	**VEGF-B**	**VEGFR-1 D2**	**PlGF**		
142 I	17 F	141 E	16 S	140 S	25 F		
143 P	18 M	142 I	17 W	141 E	26 Q		
145 I	21 Y	143 P	21 Y	142 I	29 W		
147 H	22 Q	145 I	22 T	143 P	30 G		
171 K	25 Y	172 F	48 V	145 I	33 Y		
172 F	46 I	199 Y	62 P	171 K	71 D		
173 P	48 K	202 I	63 D	172 F	74 L		
199 Y	63 D	203 G	66 L	173 P	89 L		
204 L	65 G	204 L	79 Q	199 Y	91 I		
221 L	66 L	221 L	81 L	203 G	97 P		
223 H	81 M	224 R	88 S	204 L	99 Y		
224 R	83 I		89 Q	219 N	114 P		
225 Q	86 H		90 L	221 L			
	89 Q		105 P	223 H			
	91 I			224 R			
	105 R						
	106 P						
**PDB ID: 5T89**		**PDB ID: 5T89**		**PDB ID: 5T89**		**Homology Model**	
**VEGFR-1 D3**	**VEGF-A**	**VEGFR-1 D4**	**VEGFR-1 D4**	**VEGFR-1 D5**	**VEGFR-1 D5**	**VEGFR-1 D7**	**VEGFR-1 D7**
226 T	34 D	351 R	351 R		382 R	675 S	675 S
227 N	36 F	379 K	379 K	394 D		676 S	676 S
258 L	40 P	380 S	380 S	429 Q	429 Q	677 S	677 S
259 N	41 D	381 A	381 A	431 Y	431 Y	704 E	704 E
260 T	43 I	382 R	382 R	432 E	432 E	705 P	705 P
261 R	44 E	393 K	393 K	433 K	433 K	706 G	706 G
262 V	45 Y		394 D	434 A	434 A	707 I	707 I
263 Q	46 I			435 V	435 V	708 I	708 I
264 M	63 D			436 S	436 S	717 F	717 F
277 S	64 E			437 S	437 S	719 E	719 E
278 V	65 G			438 F	438 F	720 R	720 R
279 R	84 K			439 P	439 P	722 T	722 T
280 R	85 P			451 I	451 I	725 D	725 D
282 I	86 H			453 T	453 T		
284 Q	107 K			455 T	455 T		
287 S	108 K			508 R	508 R		
290 N				510 A	510 A		
292 F				512 I	512 I		
315 S				513 E	513 E		
316 G				517 K	517 K		
317 P				519 A	519 A		
				521 T	521 T		

VEGFR-1 residues reported in the table have at least one atom within a specific distance from at least one atom of the indicated ligand. Selected thresholds were: 4.0 Å, in the case of the experimental structures of VEGFR-1 D2 in complex with VEGF-A (PDB ID: 1FLT), VEGF-B (PDB ID: 2XAC), and PlGF (PDB ID: 1RV6), which have been solved with resolution <2.8 Å; and 5.0 Å in the case of the experimental structure of VEGFR-1 D1-D6 (PDB IDs: 5T89), which has been solved with resolution = 4.0 Å, and of the molecular model built by homology for the D7 dimer, using as a template the 3D structure of VEGFR-2 D7-D7 dimer (PDB ID: 3KVQ). The latter threshold was chosen to take into account possible errors in atom positioning due to the low resolution of the structure and inherent the modeling procedure, respectively.

## Abbreviations

VEGF	vascular endothelial growth factor
VEGFR	vascular endothelial growth factor receptor
PlGF	placenta growth factor
s	soluble
Ig	immunoglubulin
HIF1α	hypoxia inducible factor 1α
FAK	focal adhesion kinase
